# Upregulation of CFTR Protects against Palmitate-Induced Endothelial Dysfunction by Enhancing Autophagic Flux

**DOI:** 10.1155/2020/8345246

**Published:** 2020-10-17

**Authors:** Hongqi Chen, Wenliang Chen, Yinlian Yao, Naobei Ye, Ning Hou, Jiandong Luo

**Affiliations:** ^1^Fifth Affiliated Hospital, Key Laboratory of Molecular Target & Clinical Pharmacology and the State Key Laboratory of Respiratory Disease, School of Pharmaceutical Sciences, Guangzhou Medical University, Guangzhou 511436, China; ^2^Department of Biology, York University, 4700 Keele Street, Toronto, ON, Canada M3J 1P3

## Abstract

Saturated free fatty acids (FFAs) elevate in metabolic symptom leading to endothelial dysfunction. Cystic fibrosis transmembrane regulator (CFTR) functionally expresses in endothelial cells. The role of CFTR in FFA-induced endothelial dysfunction remains unclear. This study is aimed at exploring the effects of CFTR on palmitate- (PA-) induced endothelial dysfunction and its underlying mechanisms. We found that PA-induced endothelial dysfunction is characterized by a decrease of cell viability, reduction of NO generation and mitochondrial membrane potential, impairment of the tube formation, but an increase of ROS generation and cell apoptosis. Simultaneously, PA decreased CFTR protein expression. CFTR agonist Forskolin upregulated CFTR protein expression and protected against PA-induced endothelial dysfunction, while CFTR knockdown exacerbated endothelial dysfunction induced by PA and blunted the protective effects of Forskolin. In addition, PA impaired autophagic flux, and autophagic flux inhibitors aggravated PA-induced endothelial apoptosis. CFTR upregulation significantly restored autophagic flux in PA-insulted endothelial cells, which was involved in increasing the protein expression of Atg16L, Atg12-Atg5 complex, cathepsin B, and cathepsin D. In contrast, CFTR knockdown significantly inhibited the effects of Forskolin on autophagic flux and the expression of the autophagy-regulated proteins. Our findings illustrate that CFTR upregulation protects against PA-induced endothelial dysfunction by improving autophagic flux and underlying mechanisms are involved in enhancing autophagic signaling mediated by the Atg16L-Atg12-Atg5 complex, cathepsin B, and cathepsin D. CFTR might serve as a novel drug target for endothelial protection in cardiovascular diseases with a characteristic of elevation of FFAs.

## 1. Introduction

Cardiovascular diseases (CVDs) are a leading cause of mortality worldwide. Saturated free fatty acids (FFAs) significantly increase in metabolic syndrome and obesity, especially in type 2 diabetes, attributing to the development of CVDs [1]. The elevation of FFAs causes endothelial dysfunction, which is an early event in the progression of CVDs [2].

Endothelium as the first barrier of vessels accounts for the regulation of vasodilation, contraction, and inflammatory response and thereby maintains normal circulatory function [3]. Elevated FFAs impair endothelium function, characterized by increased apoptosis, excessive reactive oxygen species (ROS) generation, and decreased nitric oxide (NO) production. The mechanisms underlying FFA-induced endothelial dysfunction were shown to be involved in inhibition of vascular insulin signaling and eNOS activity, excessive generation of ROS derived from NADPH oxidase and mitochondria, and activation of the NF-*κ*B signaling pathway which promotes inflammatory responses [4], whereas it remains largely unknown, and the interventive drug targets still need further research.

Autophagy is an intrinsic process maintaining cell homeostasis by degrading intracellular macromolecules and damaged organelles [5]. Generally, autophagy is classified into microautophagy, macroautophagy, and chaperone-mediated autophagy [5, 6]. In this study, autophagy is referred to as macroautophagy unless otherwise stated. Although the pathologic role of autophagy remains controversial, accumulating numbers of studies showed that autophagy protected against endothelial dysfunction induced by various conditions including oxidized low-density lipoprotein (oxLDL), hypoxia, lipopolysaccharides, and advanced glycation end products, through inhibiting inflammation and ROS generation [7–11]. In the process of autophagy, the formation of autophagosomes and functional autophagic flux is essential for degrading autophagic cargo. Impaired autophagic flux causes an accumulation of autophagy substrates such as polyubiquitinated proteins, SQSTM1/p62, and abnormal mitochondria [12]. Palmitate, a prominent component of FFAs, is widely utilized in in vitro experiments to mimic the effects of FFAs. Palmitate, in combination with high glucose impaired autophagosome formation and decreased autophagic flux participating in endothelial dysfunction [13]. Improvement of autophagic flux might serve as a critical approach to protect endothelial cells against injury induced by FFAs.

Cystic fibrosis transmembrane regulator (CFTR) is a chloride channel regulating Cl^−^ and HCO_3_^−^ cross cellular membrane. Mutation of CFTR leads to cystic fibrosis (CF) and causes severe damage to organs in the body, especially in the lungs and digestive system. CFTR widely expresses in the pulmonary epithelial cells, vascular smooth muscle cells, and vascular endothelial cells [14]. Extensive researches for the role of CFTR focus in epithelial cell function, while its role in other types of cells, such as endothelial cells, is largely unclear. A large number of studies showed that CF patients had endothelial perturbation and microvascular dysfunction [15–20], suggesting that CFTR deficiency contributes to endothelial dysfunction. Besides, CFTR was showed to protect against endothelial apoptosis from oxidative stress and inflammation [21–23]. Recently, several researches demonstrated that CFTR regulated autophagy in the intestinal cancer cells, lipopolysaccharide-induced acute lung injury, and the immune response in CF [24–26]. We thereby hypothesized that CFTR could affect FFA-induced endothelial dysfunction via the autophagy signaling pathway. In this study, we used CFTR activator and CFTR specific siRNA to investigate the effects of CFTR on palmitate-induced endothelial dysfunction and its underlying molecular mechanisms.

## 2. Materials and Methods

### 2.1. Reagents

Endothelial culture medium (ECM), fetal bovine serum (FBS), and penicillin and streptomycin (P/S) were purchased from ScienCell (California, USA). Palmitic acid and dimethyl sulfoxide (DMSO) were purchased from Sigma-Aldrich (St. Louis, MO, USA). 3-(4,5-Dimethylthiazol-2-yl)-2,5-diphenyltetrazolium bromide (MTT), diaminofluorescein-FM diacetate (DAF-FM DA), JC-1 (#C2006), and reactive oxygen detection kit (#S0033S) were purchased from Beyotime Institute of Biotechnology (Shanghai, China). The NE-PER Nuclear and Thermo Scientific Pierce BCA protein assay kit were purchased from Thermo Fisher Scientific Inc. (Waltham, MA, USA). Protease inhibitor, phosphatase inhibitor mixture, and chloroquine (CQ) were purchased from Selleck Chemicals (Houston, Texas, USA). The FITC-Annexin-V Apoptosis Detection Kit with propidium iodide (PI) and Corning Matrigel were purchased from BD Biosciences (San Jose, CA, USA). The mRFP-GFP-LC3 plasmid was purchased from Hanbio Biotechnology (Shanghai, China). Antibodies, including the Autophagy Antibody Sampler Kit (#4445), anti-lysosomal-associated membrane protein 1 (LAMP1, #9091), anti-cathepsin B (#31718), anti-cathepsin D (#2284), anti-CFTR (#78335), anti-caspase 3 (#14220), anti-cleaved caspase 3 (#9664), anti-cleaved caspase 8 (#9748), anti-caspase 8 (#9746), anti-cleaved caspase 9(#9505), and anti-caspase 9 (#9508), were obtained from Cell Signaling Technology (MA, USA). Anti-SQSTM1/p62 (#AP6006) and anti-*β*-tubulin (#BS1482M) were purchased from Bioworld company (Bloomington, MN, USA). Anti-cytochrome C (Cyt C, #AF7004) was obtained from Affinity company (Cincinnati, OH, USA). Lipofectamine RNA iMAX, Stealth RNAi™ Predesigned siRNA, and Stealth siRNA™ Negative Control were purchased from Invitrogen (Grand Island, NY, USA).

### 2.2. Cell Culture, Palmitate Preparation, and Treatment

Human umbilical vein endothelial cells (HUVECs) were cultured as our previous studies [6]. Endothelial cells were grown in ECM and maintained at 37°C in 5% CO_2_ atmosphere. Palmitic acid (PA) was dissolved in 3.3 mM bovine serum albumin to prepare the stock solution (10 mM) as described previously [6]. The pH value of the stock solution was adjusted to 7.4 with 1 M NaOH and then filtered through a 0.2 *μ*m filter. Endothelial injury was induced by treating with PA at indicated concentrations for 24 h. An equivalent vehicle (BSA) was used in the control group. In the intervention experiments, cells were pretreated with the mentioned inhibitors or activators for 45 min and then cultured with PA or vehicle for an additional 24 h, respectively.

### 2.3. MTT Assay

Cellular viability was measured using the MTT assay. HUVECs were seeded in a 96-well plate (5000 cells per well) and cultured overnight. Cells were given the indicated treatments for 24 h and then incubated with 0.5 mg/ml MTT for an additional 4 h at 37°C. The supernatant was discarded, and 100 *μ*l DMSO was added into each well to dissolve formazan crystals. The absorbance was subsequently measured at 490 nm in a multiple-function plate reader (Beckman, USA). Data were presented as the percentage of the control group.

### 2.4. Apoptosis Assay

Cell apoptosis was measured using the FITC-Annexin-V Apoptosis Detection Kit with PI according to the manufacturer's instructions. Briefly, HUVECs were digested with trypsin and suspended at a concentration of 1∗10^6^ cells/ml. Cells were centrifuged and washed with cold PBS twice and then suspended in the binding buffer. Then, cells were incubated with Annexin V-FITC and PI at room temperature (RT) for 15 min. Finally, the samples were analyzed under the BD Accuri C6 flow cytometer (BD Biosciences, USA). Early, late, and total apoptotic rates were calculated.

### 2.5. Western Blotting

HUVECs were lysed in RIPA lysis buffer supplemented with protease inhibitor and phosphatase inhibitor mixture. Protein concentration was determined by the BCA protein assay kit. Protein samples were loaded and separated in SDS-PAGE gel and transferred into a nitrocellulose membrane. The blotted membrane was incubated with the primary antibody at 4°C overnight, followed by incubation of the horseradish peroxidase-conjugated secondary antibody at RT for 1 h. Bands were developed by the ECL Plus western blotting detection system (Sangon Biotech, Shanghai, China.).

### 2.6. Analysis of Autophagic Flux

Cells were seeded into 6-well plates and loaded mRFP-GFP-LC3 adenovirus (Hanbio Biotechnology, China) with a MOI (multiplicity of infection) of 50. Then, cells were treated with indicated treatments as described in figure legends. Fluorescent images were obtained with a Leica TCSSP5 laser scanning confocal microscope equipped with a 40-times objective lens. Confocal microscopy images were binarized to black and white images by using ImageJ software to quantify the number of autophagosome (yellow) and autolysosome (red) puncta per cell.

### 2.7. Measurement of NO Generation

DAF-FM DA was used to determine NO generation according to the manufacturer's instructions. Cells were loaded with DAF-FM DA (10 *μ*M) at 37°C for 20 min. Then, fluorescent images were taken under confocal scanning microscopy. The fluorescence density was measured using ImageJ.

### 2.8. Assay of Mitochondrial Membrane Potential

JC-1 was used to determine mitochondrial membrane potential (*Δψ*m) according to the manufacturer's instructions. Cells were loaded with JC-1 at RT for 20 min. Then, the fluorescent images were taken in confocal microscopy. The ratio of green fluorescence to red fluorescence indicates the change of *Δψ*m.

### 2.9. Reactive Oxygen Species (ROS) Detection

ROS were measured using a reactive oxygen detection kit according to the manufacturer's instructions. Cells were stained with 2′,7′-dichlorodihydrofluorescein diacetate (DCFH-DA, 10 *μ*M) at 37°C for 20 min, and cell images were captured under confocal microscopy. Meanwhile, the fluorescence intensity of ROS was read at 525 nm using a multifunctional plate reader.

### 2.10. Tube Formation

Matrigel was added into 24-well plates, and it was incubated at 37°C for 1 h. Then, cells were seeded into the Matrigel-coated 24-well plate. Cells were pretreated with Forskolin and subsequently incubated with PA for 4 h. Images were taken under microscopy. The number of tubes was counted using ImageJ.

### 2.11. Statistical Analysis

Data were presented as the mean ± SD. The statistical significance between different groups was analyzed in GraphPad Prism 5.0 by using one-way ANOVA with a *post hoc* analysis of the Newman-Keuls test. *p* < 0.05 was considered statistically significant.

## 3. Results

### 3.1. PA Induced Decrease of CFTR Expression Protein in Endothelial Cells

**Figure 1:**
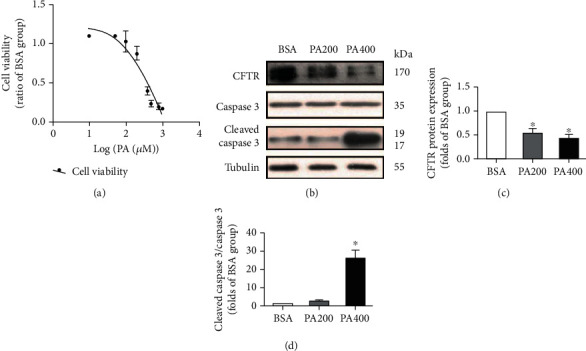
CFTR protein expression decreased in PA-induced endothelial cell injury. (a) PA induced a decrease in cell viability in HUVECs in a concentration-dependent manner. HUVECs were incubated with the indicated concentrations of PA for 24 h, and cell viability was determined by the MTT assay (*n* = 3). (b–d) Western blotting results of CFTR, caspase 3, and cleaved caspase 3 protein expression. HUVECs were incubated with PA (200 and 400 *μ*M) or vehicle (BSA) for 24 h, and then, western blot was performed. ^∗^Versus BSA group, *p* < 0.05, *n* = 4.

Firstly, we treated endothelial cells with PA to establish an endothelial dysfunction in vitro model. As shown in Figure 1(a), PA decreased the cell viability of HUVECs in a concentration-dependent manner, and IC_50_ of these effects was around 400 *μ*M, which was in line with our previous study [6]. Therefore, we used PA with a concentration of 400 *μ*M in subsequent experiments unless otherwise mentioned.

Meanwhile, we determined CFTR protein expression and the level of cleaved caspase 3. As shown in Figures 1(b)–1(d), PA significantly decreased CFTR protein expression but increased the ratio of cleaved caspase 3/caspase 3, when compared with the vehicle control group (*p* < 0.05). These results suggest that decreased expression of CFTR induced by PA is associated with endothelial injury.

### 3.2. Upregulation of CFTR Protected against PA-Induced Endothelial Cell Injury

**Figure 2:**
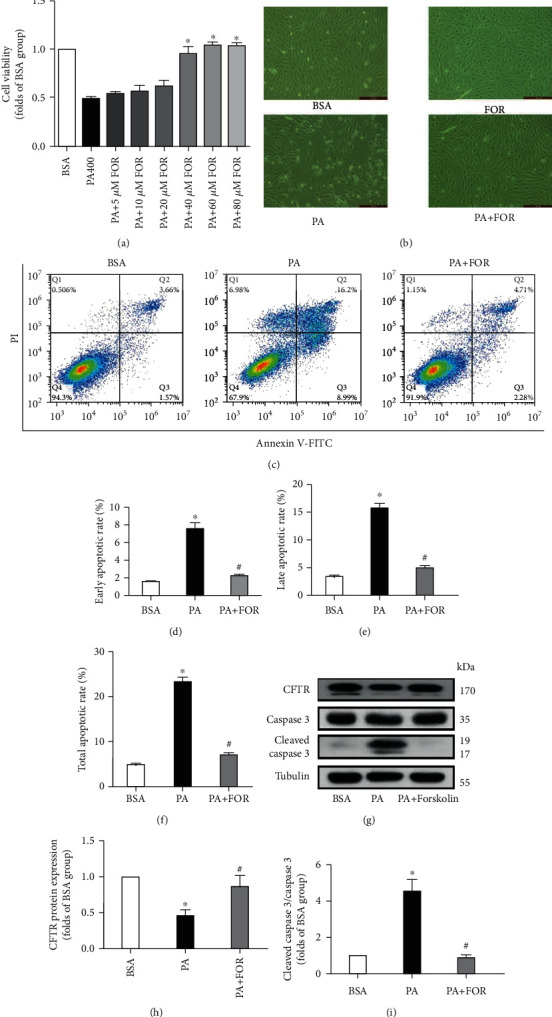
Upregulation of CFTR protected against PA-induced endothelial cell injury. (a) Forskolin protected against the decrease of cell viability in a concentration-dependent manner induced by PA in HUVECs. HUVECs were incubated with Forskolin (FOR) at various concentrations (ranging from 5 *μ*M to 80 *μ*M) for 45 min and then incubated with 400 *μ*M PA for an additional 24 h. Cell viability was determined by the MTT assay. ^∗^Versus BSA group, *p* < 0.05, *n* = 4. (b) The representative cell morphological images were shown. HUVECs were pretreated with Forskolin (60 *μ*M) for 45 min and incubated with 400 *μ*M for an additional 24 h. Then, cell images were captured randomly in the microscope (*n* = 6). (c–f) HUVECs were pretreated with Forskolin (60 *μ*M) for 45 min and then incubated with 400 *μ*M for an additional 24 h. Then, flow cytometry was carried out to measure the apoptotic rate (c). The early (d), late (e), and total (f) apoptotic rates were analyzed. ^∗^Versus BSA group; ^#^versus PA group, *p* < 0.05, *n* = 3. (g–i) Western blot results for CFTR, caspase 3, and cleaved caspase 3 expressions. Cell treatments were carried out as above. Representative western blot images were shown (g). CFTR protein expression (h) and the ratio of cleaved caspase 3/caspase 3 (i) were analyzed. ^∗^Versus BSA group; ^#^versus PA group, *p* < 0.05, *n* = 5.

Next, we used Forskolin, a CFTR agonist, to determine whether activating CFTR was involved in PA-induced endothelial injury. As shown in Figure 2(a), Forskolin significantly inhibited the PA-induced decrease of endothelial cell viability in a concentration-dependent manner. The protective effect of Forskolin reached a peak at the concentration of 60 *μ*M which totally reversed the cell injury induced by PA, and thus, this concentration was used in subsequent experiments. As shown in Figure 2(b), HUVECs in the control group showed a typical paving stone appearance, whereas PA caused a remarkable decrease of cell number with shrunken and round morphological changes. Forskolin pretreatment dramatically blocked the morphological changes of endothelial cells induced by PA, showing a similar appearance as the control group.

Besides, flow cytometry results showed that PA caused a significant increase of early, late, and total apoptotic rates in endothelial cells, which was significantly inhibited by Forskolin pretreatment (Figures 2(c)–2(f)). As shown in Figures 2(g)–2(i), Forskolin pretreatment significantly restored CFTR expression in endothelial cells compared with cells insulted with PA alone (*p* < 0.05). At the same time, Forskolin pretreatment also significantly reduced the ratio of cleaved caspase 3/caspase 3. These results together indicate that Forskolin upregulates CFTR protein expression and thus protects against PA-induced endothelial injury.

### 3.3. Forskolin Suppressed PA-Induced Apoptosis in Endothelial Cells by Increasing Mitochondrial Membrane Potential and Inhibiting ROS Generation

**Figure 3:**
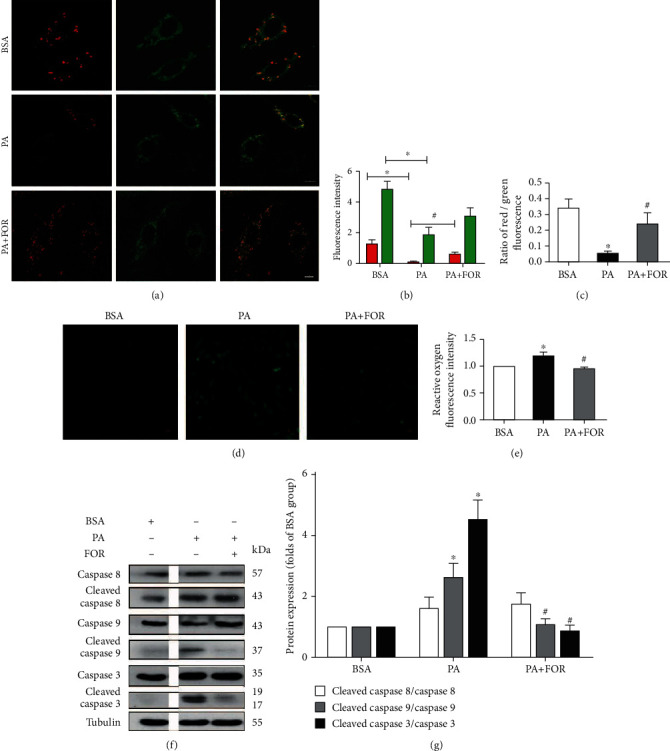
Upregulation of CFTR restored mitochondrial membrane potential and inhibited ROS generation and mitochondrial apoptotic signaling. HUVECs were pretreated with Forskolin (60 *μ*M) for 45 min and then incubated with 400 *μ*M for an additional 24 h. Then, mitochondrial membrane potential, ROS generation, and apoptotic protein expression were determined. (a) JC-1 staining was used to determine mitochondrial membrane potential. The representative images were shown. (b) The intensity of green and red fluorescence was analyzed. (c) The ratio of red/green fluorescence was analyzed. ^∗^Versus BSA group; ^#^versus PA group, *p* < 0.05, *n* = 4. (d) The representative fluorescent images of ROS were shown. (e) Fluorescence intensity was analyzed. ^∗^Versus BSA group; ^#^versus PA group, *p* < 0.05, *n* = 4. (f) The representative images of western blotting for cleaved caspase 8, cleaved caspase 9, cleaved caspase 3, caspase 3, caspase 8, and caspase 9 in HUVECs. (g) Western blot results were analyzed. ^∗^Versus BSA group; ^#^versus PA group, *p* < 0.05, *n* = 4.

PA was shown to cause cell apoptosis through impairing mitochondrial membrane potential [27]. We used JC-1 to determine mitochondrial membrane potential. JC-1 staining results showed that the ratio of red/green fluorescence intensity was significantly lower in the PA group than that in the control group, suggesting that PA induced a significant decrease of mitochondrial membrane potential in endothelial cells, whereas Forskolin pretreatment significantly attenuated the decrease of mitochondrial membrane potential induced by PA (*p* < 0.05, Figures 3(a)–3(c)).

PA creates a vicious circle between ROS generation and mitochondrial membrane potential, leading to endothelial cell apoptosis [6]. As shown in Figures 3(d) and 3(e), PA significantly increased ROS generation, displaying a higher intensity of fluorescence compared with the control group (*p* < 0.05), while ROS generation was significantly inhibited by Forskolin pretreatment (*p* < 0.05).

Next, we determined apoptotic signaling pathways underlying the protective effects of Forskolin against PA-induced endothelial apoptosis. As shown in Figures 3(f) and 3(g), PA significantly increased the ratio of cleaved caspase 9/caspase 9 and the ratio of cleaved caspase 3/caspase 3, which was significantly blocked by Forskolin pretreatment. However, PA and Forskolin pretreatment did not significantly change the ratio of cleaved caspase 8/caspase 8. These results suggest that Forskolin suppressed PA-induced endothelial apoptosis through the mitochondria-mediated apoptotic signaling pathway.

### 3.4. CFTR Knockdown Attenuated the Protective Effects of Forskolin on PA-Induced Endothelial Injury

**Figure 4:**
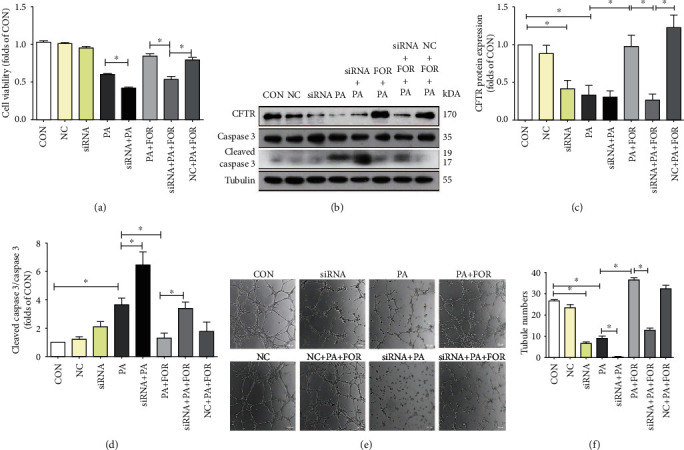
CFTR knockdown attenuated the protective effects of Forskolin on PA-induced endothelial injury. HUVECs were transfected with negative control siRNA (NC) or CFTR siRNA (siRNA) for 48 h, and then, cells were pretreated with Forskolin for 45 min, followed by the incubation of PA or vehicle for an additional 24 h for cell viability assay and western blotting or 4 h for tube formation assay. Cells were treated as the following: (1) CON group: HUVECs were incubated with BSA alone; (2) NC group: HUVECs were incubated with BSA and negative control siRNA; (3) siRNA group: HUVECs were incubated with BSA and CFTR siRNA; (4) PA: HUVECs were incubated with 400 *μ*M PA alone; (5) siRNA+PA group: HUVECs were treated with PA and CFTR siRNA; (6) PA+FOR group: HUVECs were treated with PA and 60 *μ*M Forskolin; (7) siRNA+PA+FOR group: HUVECs were treated with PA, 60 *μ*M Forskolin, and CFTR siRNA; and (8) NC+PA+FOR group: HUVECs were treated with PA, 60 *μ*M Forskolin, and negative control siRNA. (a) MTT results were shown. ^∗^*p* < 0.06, *n* = 5. (b) The representative images of western blot were shown. (c, d) Western blot results were analyzed. ^∗^*p* < 0.05, *n* = 9. (e) Representative images of tube formation were shown. (f) The number of tube formation was analyzed. ^∗^*p* < 0.05, *n* = 4.

We had observed that PA significantly reduced CFTR protein expression while Forskolin restored CFTR protein expression in PA-induced endothelial dysfunction. Hence, we silenced CFTR expression to further determine whether downregulation of CFTR participated in PA-induced endothelial dysfunction. As shown in Figure 4(a), CFTR knockdown significantly exaggerated the decrease of endothelial viability induced by PA (*p* < 0.05). Meanwhile, CFTR knockdown significantly attenuated the protective effects of Forskolin on impaired cell viability induced by PA. Besides, western blotting results (Figures 4(b)–4(d)) showed that CFTR knockdown significantly increased the ratio of cleaved caspase 3/caspase 3 in PA-insulted HUVECs when compared with cells treated with PA alone (*p* < 0.05). In addition, CFTR knockdown completely blocked the inhibitive effects of Forskolin on the ratio of cleaved caspase 3/caspase 3 (*p* < 0.05).

We next evaluated the effects of CFTR on tube formation of endothelial cells impaired by PA. As shown in Figures 4(e) and 4(f), PA significantly reduced tube formation in HUVECs; CFTR knockdown followed by PA incubation significantly decreased tube formation when compared with endothelial cells treated with PA alone (*p* < 0.05). Forskolin significantly improved tube formation of HUVECs under PA incubation, while this protective effect was completely reversed by CFTR knockdown.

### 3.5. CFTR Regulated NO Generation in HUVECs through Modifying Phosphorylation of eNOS

**Figure 5:**
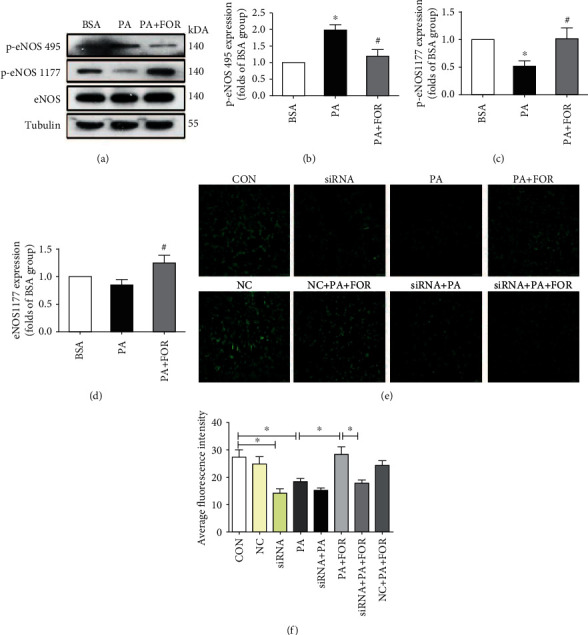
Upregulation of CFTR enhanced NO generation in HUVECs via increasing eNOS activity. (a) The representative images of western blotting were shown. HUVECs were pretreated with Forskolin for 45 min and then treated with 400 *μ*M PA for an additional 24 h. Western blot was carried out to determine the expression of p-eNOS (1177), p-eNOS (495), and eNOS. (b–d) Analysis results of western blot for p-eNOS (495), p-eNOS (1177), and eNOS, respectively. ^∗^Versus BSA group; ^#^versus PA group, *p* < 0.05, *n* = 6. (e) The representative images of NO generation were shown. (f) Analysis results of NO generation. ^∗^*p* < 0.05, *n* = 3.

In addition to excessive ROS generation, reducing NO generation also contributes to endothelial dysfunction. Endothelial nitric oxide synthase (eNOS) is a key enzyme for NO production. Phosphorylation at the site of Ser-1177 activates the activity of eNOS, while phosphorylation at the site of Thr-495 deactivates the enzyme activity. As shown in Figures 5(a)–5(d), PA significantly increased phosphorylation of eNOS at Thr-495 but decreased phosphorylation of Ser-1177. Forskolin pretreatment significantly inhibited these changes induced by PA. In line with the western blotting results, as shown in Figures 5(e) and 5(f), PA significantly reduced NO generation in HUVECs, while Forskolin treatment significantly restored the NO generation (*p* < 0.05), whereas CFTR knockdown significantly attenuated the effects of Forskolin on NO generation under PA incubation.

### 3.6. Effects of CFTR on Autophagic Flux in Endothelial Cells

**Figure 6:**
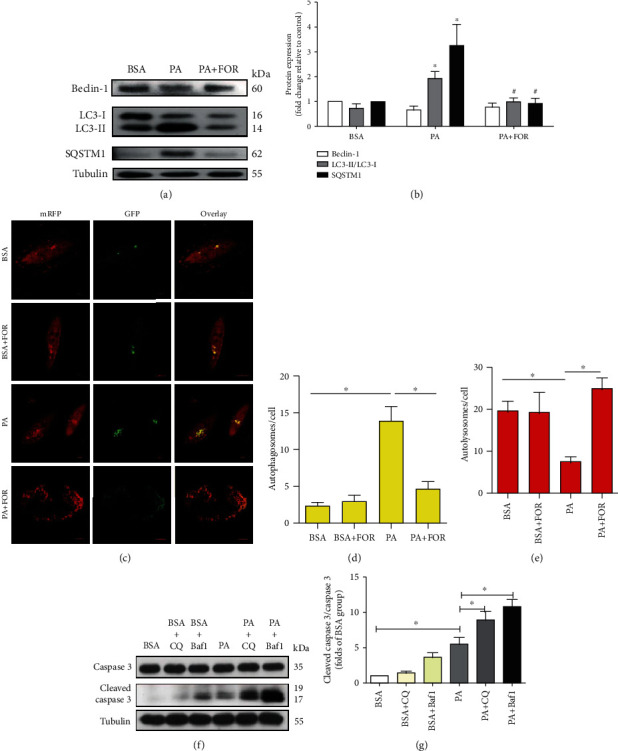
Upregulation of CFTR protected against endothelial apoptosis induced by PA via improving autophagic flux. (a) The representative images of western blotting for the expression of beclin-1, SQSTM1, LC3-II, and LC3-I in HUVECs. HUVECs were pretreated with Forskolin for 45 min and then treated with 400 *μ*M PA for an additional 24 h. Western blot was carried out to determine the expression of autophagic proteins. (b) Analysis results of western blot were shown. ^∗^Versus BSA group; ^#^versus PA group, *p* < 0.05, *n* = 3. (c) The representative images of mRFP-GFP-LC3 fluorescence measuring the autophagic flux in HUVECs. (d, e) The analysis results of mRFP-GFP-LC3 fluorescence were shown. ^∗^^,#^*p* < 0.05, *n* = 3. (f) The representative images of western blotting for the expression of cleaved caspase 3 and caspase 3 in HUVECs. HUVECs were pretreated with CQ or Baf1 for 45 min and then treated with 400 *μ*M PA or equivalent concentration of vehicle for an additional 24 h. Then, western blot was carried out. (g) Analysis results of western blot were shown. ^∗^*p* < 0.05, *n* = 4.

We next investigated if autophagy is a mechanism underlying the protective effects of CFTR against PA-induced endothelial dysfunction. As Figures 6(a) and 6(b) show, PA insult significantly increased the ratio of LC3-II/LC3-I protein and SQSTM1 in endothelial cells when compared with the vehicle control group, which was significantly inhibited by Forskolin pretreatment (*p* < 0.05). Beclin-1 protein expression was not significantly different between these groups. These results suggest that PA leads to impaired autophagic flux.

We next used the tandem fluorescent-tagged LC3 (mRFP-GFP-LC3) to further determine the effects of Forskolin on autophagic flux. The yellow and red puncta in the overlay images represent the autophagosomes and autolysosomes, respectively [28]. As shown in Figures 6(c)–6(e), we found that PA induced significant increases in the number of autophagosomes but decreased the number of autolysosomes in endothelial cells, while Forskolin pretreatment significantly inhibited these changes. These results suggest that Forskolin improves the autophagic flux in HUVECs under PA incubation.

Chloroquine (CQ) and Bafilomycin A1 (Baf1) were autophagic flux inhibitors. As shown in Figures 6(f) and 6(g), both CQ and Baf1 treatment significantly increased the ratio of cleaved caspase 3/caspase 3 in PA-insulted endothelial cells when compared with PA incubation alone, suggesting that blockage of autophagic flux attributes to PA-induced endothelial apoptosis.

The above results suggest that upregulation of CFTR by Forskolin suppresses PA-induced endothelial dysfunction by improving autophagic flux.

### 3.7. CFTR Regulated the Expressions of Atg16L and Atg12-Atg5 Complex

**Figure 7:**
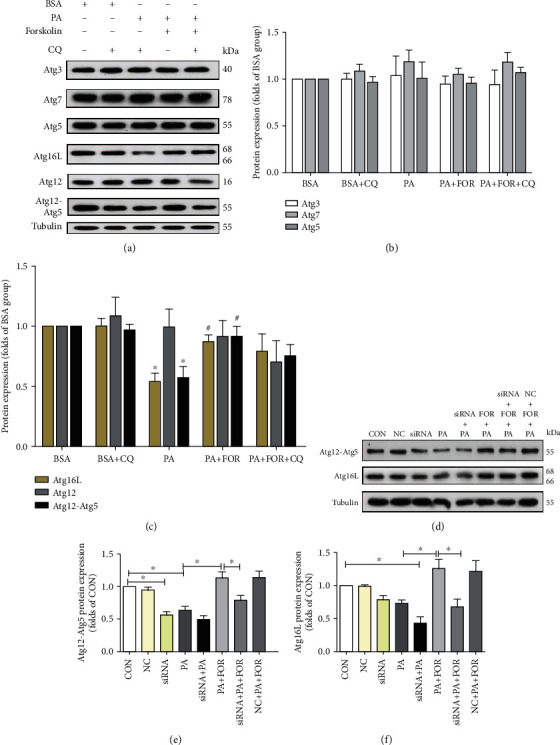
Upregulation of CFTR improved autophagic flux by increasing the expression of Atg16L and Atg12-Atg5 complex. (a) The representative images of western blotting for the expression of Atgs in HUVECs. HUVECs were pretreated with Forskolin and CQ (20 *μ*M) for 45 min and then treated with PA for an additional 24 h followed by western blot. (b, c) Analysis result of western blot of Atgs. ^∗^Versus BSA group; ^#^versus PA group, *p* < 0.05, *n* = 8. (d) The representative images of western blot for the expression of Atg12-Atg5 and Atg16L in HUVECs with CFTR knockdown. HUVECs were transfected with negative control siRNA or CFTR siRNA for 48 h and then treated with PA for an additional 24 h. Western blot was carried out to determine the expression of Atg12-Atg5 and Atg16L. (e, f) Analysis results of western blot for Atg12-Atg5 and Atg16L. ^∗^*p* < 0.05, *n* = 10.

Next, we explored the mechanism by which CFTR improved the autophagic flux. Atg (the autophagy-related proteins) regulated autophagosome formation and the fusion with lysosome [29–34]. As shown in Figures 7(a)–7(c), PA did not significantly affect the expression of Atg3, Atg5, Atg7, and Atg12 in endothelial cells but significantly reduced Atg16L expression and the level of Atg12-Atg5 complex compared with the vehicle group (*p* < 0.05). Forskolin pretreatment significantly increased the levels of Atg16L and Atg12-Atg5 complex in PA-insulted endothelial cells, whereas CFTR knockdown significantly attenuated the increase of Atg16L and Atg12-Atg5 complex resulting from Forskolin pretreatment in PA-insulted endothelial cells (Figures 7(d)–7(f)). CFTR knockdown alone also significantly decreased the expression of the Atg12-Atg5 complex. These results implicate that CFTR upregulation improves autophagic flux by promoting the expression of Atg16L and Atg12-Atg5 complex, without affecting Atg3/5/7/12.

### 3.8. CFTR Regulated the Expression of Cathepsin B and Cathepsin D

**Figure 8:**
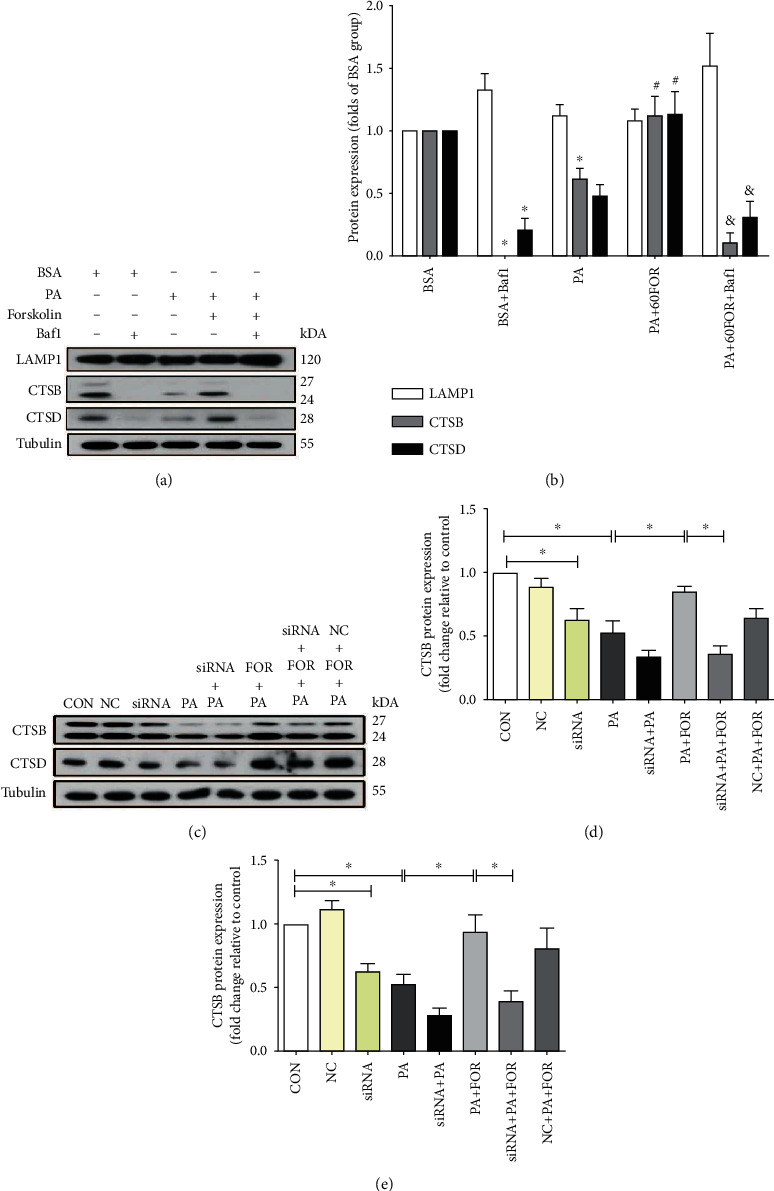
Upregulation of CFTR improved autophagic flux by increasing the protein expression of cathepsin B and cathepsin D. (a) The representative images of western blotting for the expression of LAMP1, CTSD, and CTSB in HUVECs. HUVECs were pretreated with Forskolin and Baf1 (20 nM) for 45 min and then treated with PA for an additional 24 h followed by western blot. (b) The analysis of western blot. ^∗^Versus BSA group; ^#^versus PA group; ^&^versus PA+FOR group, *p* < 0.05, *n* = 6. (c) The representative images of western blotting for the expression of CTSD and CTSB in HUVECs with CFTR knockdown. HUVECs were transfected with negative control siRNA or CFTR siRNA for 48 h and then treated with PA for an additional 24 h followed by western blot. (d, e) Analysis results of western blot. ^∗^*p* < 0.05, *n* = 10.

Lysosomes are essential organelles for macromolecular clearance, playing a key role in the final formation and function of autolysosome. The lysosomal membrane protein LAMP1 is responsible for maintaining lysosomal integrity and pH [35]. Cathepsin B (CTSB) and cathepsin D (CTSD), as lysosomal cysteine proteases, regulate the degradation or processing of lysosomal proteins and thus regulate autophagic flux [36]. The autophagy inhibitor Bafilomycin A1 (Baf1) can effectively inhibit the proton pump on the lysosomal membrane, increase the lysosomal pH, and inhibit the function of lysosomes. As shown in Figures 8(a) and 8(b), we observed that PA significantly reduced the expression of CTSD and CTSB protein without changing the expression of LAMP1 protein. Baf1 had a similar effect as PA did. Forskolin pretreatment significantly restored the expression of CTSD and CTSB in PA-insulted endothelial cells. In contrast, CFTR knockdown alone decreased CTSD and CTSB expression when compared with the negative siRNA group (Figures 8(c)–8(e)). Moreover, the increases of both CTSD and CTSB caused by Forskolin were significantly inhibited by CFTR knockdown in endothelial cells under PA incubation. Therefore, these results indicate that CFTR upregulation by Forskolin increases the expression of CTSB and STSD which can promote autophagic degradation in lysosomes.

## 4. Discussion

FFAs elevate in metabolic syndromes, such as obesity and type 2 diabetes mellitus (T2DM), leading to endothelial dysfunction, which occurs in the early stages of CVDs [2]. Endothelial dysfunction is defined as endothelial apoptosis, excessive ROS generation, and insufficient NO function [4]. The molecular mechanisms involved in FFA-induced endothelial dysfunction remain largely unknown. PA is one of the prominent components of FFAs, which is widely used to mimic the effects of FFAs in in vitro experiments [6, 37]. In this study, we found that (1) PA significantly caused a significant decrease of CFTR expression in endothelial cells; (2) Forskolin significantly restored CFTR protein expression and inhibited PA-induced decrease of cellular viability and apoptosis in endothelial cells by inhibiting the mitochondria-mediated apoptotic pathway; (3) CFTR upregulation by Forskolin restored the balance between ROS and NO generation and increased tube formation in PA-insulted endothelial cells; (4) CFTR upregulation significantly improved autophagic flux in endothelial cells impaired by PA, which was involved in the restoration of the Atg12-Atg5 complex, Atg16L protein, CTSB, and CTSD protein expression; and (5) CFTR knockdown aggravated endothelial cell injury induced by PA and suppressed the protective effects of Forskolin against endothelial injury.

PA induced oxidative stress, inflammatory response, and calcium overload, and these harmful factors ultimately resulted in endothelial apoptosis [6, 12, 27]. Our previous study revealed that potassium ion channel Kv1.5 overexpression mediated endothelial dysfunction induced by PA [6]. Here, we further investigate if other ion channels participate in PA-induced endothelial dysfunction. CFTR is an ATP-gated Cl^−^ channel, and downregulation of CFTR causes apoptosis via ROS and inflammation in renal cells, vascular smooth muscle cells, and other cell types [23, 38–40]. In this study, we found that CFTR expression decreased in PA-insulted endothelial cells; Forskolin significantly inhibited PA-induced endothelial dysfunction showing as increasing cell viability, inhibiting cell apoptosis, reducing ROS generation, and restoring NO generation. Forskolin inhibited PA-induced apoptosis in HUVECs by reducing ROS generation via both cAMP/protein kinase A (PKA) and the AMP-activated protein kinase (AMPK) signaling pathways [12]. Both PKA and AMPK regulate CFTR activity with an opposite effect [41]. Besides, Forskolin not only rapidly increases CFTR channel activity but also promotes CFTR gene transcription and protein expression in long-term (over 18 h) incubations [42–44]. This study showed that Forskolin incubation for 24 h significantly restored CFTR protein expression in PA-stimulated endothelial cells, implying that Forskolin might increase CFTR expression through increasing CFTR gene transcription. However, the molecular mechanisms underlying PA-induced reduction of CFTR protein expression in endothelial cells remain unclear, which might be involving inhibition of gene transcription and increase of protein degradation. Future researches are needed to illustrate the regulated mechanisms of CFTR expression by Forskolin in PA-insulted endothelial cells.

Whether CFTR regulated by Forskolin is involved in the protective effects of Forskolin against PA-induced endothelial dysfunction remains unclear. Our results showed that Forskolin significantly restored CFTR protein expression in PA-insulted endothelial cells while CFTR knockdown significantly attenuated the protective effects of Forskolin against PA-induced endothelial dysfunction. Moreover, CFTR knockdown also aggravated the endothelial injury induced by PA. Therefore, our findings demonstrate that CFTR upregulation by Forskolin suppresses PA-induced endothelial dysfunction.

Cell apoptosis is mainly mediated by death receptor- and mitochondria-mediated apoptotic signaling pathways [45]. Death receptors bind to their ligands; then, the Fas-associated death domain (FADD) protein is recruited to the death domain, subsequently resulting in caspase 8 recruitment and activation, which finally triggers the apoptotic caspase cascade [45]. The mitochondrial apoptotic signaling pathway is associated with mitochondrial dysfunction: decreased mitochondrial membrane potential, and proapoptotic Bcl-2 family member translocation from cytosol to mitochondria leading to the release of Cyt C, and subsequently causes the activation of caspase 9 apoptotic caspase cascade [45]. It was reported that PA induced endothelial cell apoptosis by stimulating the death receptor pathway mediated by the TNF-R1/TNFR1-associated death domain protein (TRADD)/caspase 8 pathway [46], whereas most studies showed that PA led to mitochondrial dysfunction and increased the protein levels of Bax, Cyt C, and cleaved caspase 9 but reduced Bcl-2 protein expression [13, 47–49]. In our previous study, we found that PA induced apoptosis in HUVECs through the mitochondria-mediated signaling pathway showing as a decrease of mitochondrial membrane potential and the ratio of Bcl-2/Bax [6]. This study was in line with our previous study, showing that PA induced a decrease of mitochondrial membrane potential but an increase of the ratio of cleaved caspase 9/caspase 9, without a significant change of the ratio of cleaved caspase 8/caspase 8. Furthermore, our study showed that Forskolin pretreatment inhibited the ratio of cleaved caspase 9/caspase 9 while restoring mitochondrial membrane potential, demonstrating that CFTR upregulation inhibited PA-induced endothelial apoptosis mediated by the mitochondrial apoptotic signaling pathway.

PA-induced endothelial dysfunction is associated with excessive ROS generation and insufficient NO generation. In line with other researchers' results [12], our results showed that CFTR upregulation suppressed PA-induced ROS generation but enhanced NO generation. Endothelial NO is regulated by eNOS, the activity of which is inactivated or activated by the phosphorylation of eNOS at the site of Thr-495 or Ser-1177, respectively. Our results showed that Forskolin increased the p-eNOS (Ser-177) level, while it reduced the level of p-eNOS (Thr-495) in endothelial cells incubated with PA. In contrast, CFTR knockdown significantly attenuated the effect of NO generation restored by Forskolin. These findings suggest that CFTR upregulation activates eNOS to increase endothelial NO generation. Suppression of excessive ROS generation and increase of NO generation can inhibit endothelial cell apoptosis [12, 48]. Our results demonstrated that CFTR upregulation restored the balance between ROS and NO generation and thus inhibit PA-induced endothelial injury.

Autophagy is a conserved mechanism responsible for the degradation of proteins and the clearance of damaged organelles in autolysosomes: maintaining the cellular homeostasis [5]. Beclin-1 is required for the formation of the nucleation complex, forming a transient complex with Atg14, vacuolar protein sorting 15 (vps15), and the vps34. LC3 is the most commonly accepted autophagosome marker with two forms: the soluble cytosolic LC3-I and the autophagosome-bound LC3-II [50]. SQSTM1/p62 is widely used as an indicator of autophagic flux in combination with LC3-II turnover [51]. We found that PA did not affect the beclin-1 expression but significantly increased the ratio of LC3-II/LC3-I and SQSTM1/p62 protein expression, indicating that PA-induced abnormal autophagy does not depend on beclin-1 signaling and PA stimulation causes a blockage of autophagic flux in endothelial cells. The defect of autophagy flux leads to endothelial cell apoptosis [52]. In contrast, restoring the functional autophagy protects against endothelial dysfunction induced by various stimuli [11]. Our study also showed that both autophagy flux inhibitors, CQ and Baf1, aggravated PA-induced endothelial apoptosis suggesting that the blockage of autophagy flux participated in PA-induced endothelial dysfunction.

CFTR has been reported as an important tumor suppressor gene by inhibiting autophagy [24]. CFTR mediates autophagy via CFTR-beclin-1 signaling [53, 54]. However, it remains unknown whether and how CFTR regulated autophagy in endothelial cells under PA stimulus. Our results showed that CFTR upregulation by Forskolin significantly decreased the ratio of LC3-II/LC3-I and SQSTM1/p62 protein expression in endothelial cells incubated with PA, whereas these effects were reversed by CFTR knockdown. We further evaluated the effects of CFTR activation on autophagic flux by using the mRFP-GFP-LC3 fluorescence assay, and the results together demonstrate that CFTR upregulation improves autophagic influx in endothelial cells insulted by PA.

The defects of autophagic function can occur at two different phases: (1) the phase of formation of autophagosomes or (2) the fusion stage with lysosomes impairing the degradation function [5]. More than 30 Atg genes control the process of autophagy, including the initiation of autophagy, the nucleation complex generation, autophagosome formation, and cargo identification [55]. Two ubiquitin-like conjugation systems participate in membrane elongation and autophagosome formation in autophagy: Atg12-Atg5 system and LC3-PE (phosphatidylethanolamine) conjugation system [56]. In the Atg12-Atg5 system, Atg7 activates Atg12 to induce Atg12-Atg5 conjugate, and then, Atg12-Atg5 and Atg16L form a protein complex. On the other hand, Atg7 activates LC3 via the E2 enzyme Atg3, transforming LC3-I to form LC3-II-PE conjugate [57]. Our results showed that PA decreased Atg16L and Atg12-Atg5 complex protein expression in endothelial cells but did not significantly affected Atg3, Atg7, Atg5, and Atg12 protein expression, suggesting that PA suppresses Atg12-Atg5-Atg16L complex formation in endothelial cells. We further showed that CFTR upregulation by Forskolin significantly restored Atg16L and Atg12-Atg5 complex protein expression in endothelial cells under PA incubation, whereas these effects were largely attenuated by CFTR knockdown, indicating that CFTR upregulation enhances Atg12-Atg5 signaling which favors the membrane elongation during the process of autophagosome formation.

CTSB and CTSD are the essential proteolytic enzymes accounting for the lysosomal protein degradation. Inhibition of CTSB impaired autophagic flux and increased the SQSTM1/p62 protein level [35]. LAMP1 is one of the lysosomal membrane proteins attributing to maintaining the integrity of the lysosomal membrane [58]. Our results showed that LAMP1 expression was not affected by PA. We found that PA decreased the protein expression of both CTSB and CTSD, while Forskolin significantly restored their expressions. In parallel, autophagic flux inhibitor Bafilomycin A1 reduced CTSB and CTSD protein expression and restrained the changes of CTSB and CTSD induced by Forskolin. In addition, CFTR knockdown significantly reversed the effects of Forskolin on the expressions of CSTD and CTSB. These results demonstrate that CFTR upregulation improves the expressions of CSTD and CTSB, which contribute to enhancing autophagic flux by increasing the degradation ability of autolysosomes.

## 5. Conclusions

**Figure 9:**
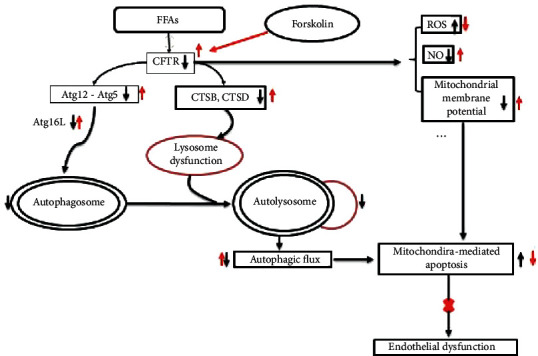
Schematic diagram for the effects of CFTR upregulation on PA-induced endothelial dysfunction. PA decreased CFTR protein expression and induced endothelial dysfunction: increasing ROS production, decreasing NO generation, reducing mitochondrial membrane potential, and inducing mitochondria-mediated apoptosis (black arrows). Upregulation of CFTR by Forskolin significantly blocked the harmful effects of PA on endothelial cells through improving autophagic flux with an underlying mechanism being involved in enhancing Atg12-Atg5-Atg16L complex formation and restoring the CTSB and CTSD protein expressions (red arrows).

A schematic diagram for the main findings of this study is shown in Figure 9. Taken together, this study demonstrates that CFTR upregulation protects against PA-induced endothelial dysfunction through improving autophagic flux with an underlying mechanism being involved in enhancing Atg12-Atg5-Atg16L complex formation and restoring the CTSB and CTSD protein expression. CFTR might serve as a novel target for endothelial protection in cardiovascular diseases characterized by elevation of FFAs.

## Data Availability

The data used to support the findings of this study are available from the corresponding authors upon request.
